# Prevalence and Predictors of Bone and Ocular Complications in Children With Steroid-Sensitive Nephrotic Syndrome: A Cross-Sectional Study

**DOI:** 10.7759/cureus.109153

**Published:** 2026-05-18

**Authors:** Kajal Patel, Mritunjay Kumar, Swarastra P Singh, Gaurav K Upadhyaya, Rashmi Kumari

**Affiliations:** 1 Paediatrics, All India Institute of Medical Sciences, Raebareli, Raebareli, IND; 2 Ophthalmology, All India Institute of Medical Sciences, Raebareli, Raebareli, IND; 3 Orthopaedic Surgery, All India Institute of Medical Sciences, Raebareli, Raebareli, IND; 4 Preventive Medicine, Dr. Ram Manohar Lohia Institute of Medical Sciences, Lucknow, IND

**Keywords:** cataract, children, nephrotic syndrome, osteopenia, steroid

## Abstract

Objective

The main objective of this study is to assess bone mineral density (BMD) and ocular complications in children with steroid-sensitive nephrotic syndrome (SSNS) receiving prolonged corticosteroid therapy and to identify factors associated with these complications.

Methods

This cross-sectional observational study was conducted at a tertiary care center over 18 months. Children aged 1-18 years with SSNS who had received corticosteroids for more than six months were enrolled. Clinical details, including cumulative steroid dose and duration of therapy, were recorded. All participants underwent ophthalmologic evaluation, biochemical assessment, and lumbar spine dual-energy X-ray absorptiometry (DEXA) to determine BMD Z-scores. Associations between steroid exposure and bone and ocular outcomes were analyzed.

Results

Fifty children were included, 66% were boys, and the mean age was 6.08 ± 2.84 years. Osteopenia was found in 25 children (50%), and osteoporosis in nine (6%). Longer steroid duration was significantly associated with lower BMD (p = 0.025). Children with osteoporosis had significantly higher parathormone (PTH) and alkaline phosphatase (ALP) levels and lower vitamin D levels (p < 0.001). Ocular complications were identified in nine children (18%), most commonly refractive errors (16%). Refractive errors were significantly associated with longer steroid duration (p = 0.013) and higher cumulative steroid dose (p = 0.012). Posterior subcapsular cataract and raised intraocular pressure were observed in one child each.

Conclusion

Prolonged corticosteroid therapy in children with SSNS is associated with reduced BMD and ocular complications. Regular monitoring of bone health, vitamin D status, and ophthalmologic evaluation should be part of long-term care.

## Introduction

Nephrotic syndrome (NS) is the most common glomerular disease in school-aged and adolescent children, with an incidence ranging from 1.15 to 16.9 per 100,000 child population, varying by region and ethnicity [1]. It is characterized by heavy proteinuria, hypoalbuminemia, and oedema [2]. About 90% of children achieve remission with corticosteroids within the first four weeks, but a substantial proportion experience frequent relapses requiring repeated steroid courses [3].

Prolonged corticosteroid exposure is associated with multiple systemic adverse effects, including endocrine, metabolic, musculoskeletal, and ocular complications such as obesity, hypertension, cataracts, and osteoporosis [4]. Children are particularly vulnerable to steroid-induced bone loss, with impaired mineralization, reduced bone density, and growth retardation [5,6]. Previous studies have shown that reduced bone mineral density (BMD) is common in children with idiopathic NS receiving prolonged steroid therapy [6]. In Indian children, the adverse skeletal effects of corticosteroids may be further accentuated by the high prevalence of vitamin D deficiency and suboptimal dietary calcium intake.

Recent studies suggest that refractive errors are the most frequent ocular abnormality in children with NS receiving prolonged corticosteroid therapy [7]. Steroid therapy may also induce ocular complications, including posterior subcapsular cataract, raised intraocular pressure, and refractive errors [8]. Despite these recognized risks, standardized recommendations for routine bone and ocular monitoring in children on long-term corticosteroids are limited.

This study aimed to evaluate BMD, biochemical markers (calcium, vitamin D, parathormone (PTH), and alkaline phosphatase (ALP)), and ocular changes in children with steroid-sensitive nephrotic syndrome (SSNS) receiving prolonged corticosteroid therapy and to identify clinical and treatment-related factors associated with these complications. Most previous studies have evaluated either skeletal or ocular complications separately, and few have assessed both systems comprehensively in the same cohort. Furthermore, standardized recommendations for routine bone and ophthalmologic monitoring in children receiving long-term corticosteroid therapy remain limited.

## Materials and methods

Study design and setting

This cross-sectional observational study was conducted in the Department of Pediatrics, in collaboration with the Departments of Ophthalmology and Orthopaedics, at the All India Institute of Medical Sciences (AIIMS), Raebareli, Uttar Pradesh, India. The study was carried out over 18 months (July 2023 to December 2024).

Study participants

Children aged 1-18 years with SSNS attending the pediatric outpatient department were screened for eligibility. SSNS was defined by nephrotic-range proteinuria (>50 mg/kg/d), with hypoalbuminemia (<30 g/L) and documented response to corticosteroids. Inclusion criteria were age 1-18 years, diagnosis of SSNS, and treatment with systemic corticosteroids for more than six months. Exclusion criteria were pre-existing ocular or bone disease prior to the diagnosis of NS, secondary NS, steroid-resistant NS, and refusal or inability to provide informed consent. As this was a time-bound cross-sectional study, all eligible children fulfilling the inclusion criteria during the study period were included. Hence, a formal sample size calculation was not performed, and a total of 50 participants were enrolled using consecutive sampling.

Data collection and measurements

Data were recorded using a structured proforma. Demographic and clinical details included age, sex, anthropometry, age at onset of NS, duration of illness, family history, NS subtype, number of relapses, and comorbidities. Cumulative steroid dose and duration of therapy were calculated from treatment records. Participants were classified as infrequently relapsing NS (IFRNS), frequently relapsing NS (FRNS), or steroid-dependent NS (SDNS) as per the revised Indian Society of Pediatric Nephrology (ISPN) guidelines for SSNS [9].

Based on disease activity at the time of enrolment, participants were categorized into two groups for exploratory subgroup analysis: Group 1: Children in remission at the time of assessment; Group 2: Children with active relapse, defined by urine protein/creatinine ratio >2.

Blood samples were obtained for serum calcium, phosphorus, magnesium, albumin, creatinine, cholesterol, ALP, PTH, and 25-hydroxy vitamin D. Urinary protein and creatinine were measured, and the protein/creatinine ratio was calculated. For children in relapse, investigations were performed during active disease, while for children in remission, results from the most recent relapse were retrieved from medical records.

All participants underwent a comprehensive ophthalmological examination, including visual acuity assessment (Snellen chart for children >8 years and Cardiff test for those <8 years), external ocular examination, slit-lamp biomicroscopy for anterior segment evaluation, intraocular pressure measurement using non-contact tonometry, confirmed by Goldmann applanation tonometry, and posterior segment examination by direct and indirect ophthalmoscopy. Optical coherence tomography was performed where central serous chorioretinopathy was suspected. Ocular outcomes assessed included refractive errors, posterior subcapsular cataract, raised intraocular pressure, steroid-induced glaucoma, and exophthalmos.

BMD was measured using dual-energy X-ray absorptiometry (DEXA) at the lumbar spine (L2-L4). Results were expressed as age- and sex-adjusted Z-scores. Osteopenia was defined as a Z-score ≤ -1 SD and osteoporosis as a Z-score ≤ -2.5 SD, in accordance with World Health Organization and International Society for Clinical Densitometry guidelines.

Total cumulative duration of corticosteroid therapy (years) and cumulative oral prednisolone dose (mg) were calculated from treatment records.

Ethical consideration

The study was approved by the Institutional Ethics Committee (IEC approval number: 2024-20-PGTH-7) and conducted in accordance with the Declaration of Helsinki. Written informed consent and assent were obtained.

Statistical analysis

Data were analyzed using Jamovi version 2.3.38 (Jamovi Project, University of Sydney, Australia). Continuous variables were expressed as mean ± standard deviation or median (interquartile range), and categorical variables as frequencies and percentages. Group comparisons were performed using an independent t-test for two groups and a one-way analysis of variance (ANOVA) for more than two groups. Non-parametric tests (Mann-Whitney U test or Kruskal-Wallis test) were used where appropriate. Categorical variables were compared using chi-square or Fisher’s exact test. Pearson correlation was used to assess relationships between variables. A p-value <0.05 was considered statistically significant.

## Results

Fifty children with idiopathic SSNS were enrolled; 33 (66%) were boys and 17 (34%) were girls, with a mean (SD) age of 6.08 (2.84) years (Table 1). Based on disease activity at enrolment, 41 (82%) were in remission (Group 1) and nine (18%) had active relapse (Group 2). Subtypes included FRNS in 25 (50%), IFRNS in 15 (30%), and SDNS in 10 (20%). The mean cumulative steroid duration was 1.31 (0.97) years, and the cumulative dose was 4906.94 (2403.94) mg. Biochemical parameters showed mean serum calcium 9.44 (0.99) mg/dL, phosphorus 5.71 (0.85) mg/dL, PTH 42.76 (15.77) pg/mL, ALP 113.30 (73.46) U/L, and 25-hydroxy vitamin D 20.56 (6.44) ng/mL.

**Table 1:** Baseline characteristics of study population (n = 50) NS: nephrotic syndrome; PTH: parathormone; ALP: alkaline phosphatase

Variable	Value
Demographics	
Age, years (mean ± SD)	6.08 ± 2.84
Male, n (%)	33 (66)
Disease classification, n (%)	
Frequently relapsing NS	25 (50)
Infrequently relapsing NS	15 (30)
Steroid-dependent NS	10 (20)
Disease activity, n (%)	
Remission (Group 1)	41 (82)
Active relapse (Group 2)	9 (18)
Steroid exposure	
Cumulative duration, years (mean ± SD)	1.31 ± 0.97
Cumulative dose, mg (mean ± SD)	4906.94 ± 2403.94
Dose per kg body weight, mg/kg (mean ± SD)	274.27 ± 189.68
Biochemical parameters	
Serum calcium, mg/dL (mean ± SD)	9.44 ± 0.99
Serum phosphorus, mg/dL (mean ± SD)	5.71 ± 0.85
PTH, pg/mL (mean ± SD)	42.76 ± 15.77
ALP, U/L (mean ± SD)	113.30 ± 73.46
25-OH vitamin D, ng/mL (mean ± SD)	20.56 ± 6.44

DXA evaluation of the lumbar spine showed normal BMD in 22 (44%), osteopenia in 25 (50%), and osteoporosis in three (6%) children (Table 2). The overall mean BMD Z-score was -1.37 ± 0.92. The mean Z-scores in children with normal BMD, osteopenia, and osteoporosis were -0.57 ± 0.49, -1.6 ± 0.42, and -3.04 ± 0.7, respectively. Cumulative duration of steroid therapy differed significantly across BMD categories (F = 3.985, p = 0.025), whereas cumulative steroid dose did not show a significant difference (F = 0.56, p = 0.575). Children with osteoporosis had markedly higher PTH (88.00 ± 10.00 vs 41.46 ± 10.73 pg/mL) and ALP (393.00 ± 4.58 vs 97.36 ± 30.14 U/L), and lower vitamin D levels, compared to those with normal BMD (F = 29.22 and 247.99, respectively; p < 0.001 for both). Serum calcium was not significantly associated with BMD categories (F = 0.892, p = 0.417). Osteoporosis was observed only in children aged one to six years, whereas osteopenia and normal BMD were distributed across older age groups, without significant differences by age or NS subtype.

**Table 2:** BMD assessment and associated factors (n = 50) Values expressed as mean ± SD. BMD: bone mineral density; PTH: parathyroid hormone; ALP: alkaline phosphatase; ANOVA: analysis of variance

Variable	Normal BMD (n = 22)	Osteopenia (n = 25)	Osteoporosis (n = 3)	F-value	p-value	Statistical test
Age, years	5.73 ± 2.23	6.56 ± 3.37	4.67 ± 1.53	0.892	0.417	One-way ANOVA
Cumulative steroid duration, years	0.93 ± 0.62	1.58 ± 1.06	2.00 ± 1.32	3.985	0.025	One-way ANOVA
Cumulative steroid dose, mg	4527.73 ± 2252.95	5194.80 ± 2428.24	5553.33 ± 3422.36	0.56	0.575	One-way ANOVA
Steroid dose, mg/kg	236.77 ± 81.94	304.79 ± 249.01	355.65 ± 168.65	1.024	0.367	One-way ANOVA
PTH, pg/mL	41.46 ± 10.73	38.52 ± 10.63	88.00 ± 10.00	29.22	<0.001	One-way ANOVA
ALP, U/L	97.36 ± 30.14	93.56 ± 13.81	393.00 ± 4.58	247.985	<0.001	One-way ANOVA
25-OH vitamin D, ng/mL	21.82 ± 6.01	19.92 ± 6.58	15.00 ± 7.21	11.363	<0.001	One-way ANOVA

Cumulative steroid duration and cumulative steroid dose differed significantly among NS subtypes (F = 7.30, p = 0.002, and F = 5.53, p = 0.007, respectively; Table 3). Mean vitamin D levels were lower in SDNS (16.90 ± 7.16 ng/mL) compared to FRNS (20.72 ± 6.64 ng/mL) and IFRNS (22.73 ± 4.42 ng/mL), although this difference did not reach statistical significance (F = 2.70, p = 0.078). There were no significant differences in calcium, phosphorus, PTH, or ALP levels among the three subtypes.

**Table 3:** Steroid exposure and biochemical profile in nephrotic syndrome subtypes Values expressed as mean ± SD. FRNS: frequently relapsing nephrotic syndrome; IFRNS: infrequently relapsing nephrotic syndrome; SDNS: steroid-dependent nephrotic syndrome; ANOVA: analysis of variance; ALP: alkaline phosphatase; PTH: parathormone

Variable	FRNS (n = 25)	IFRNS (n = 15)	SDNS (n = 10)	F-value	p-value	Statistical test
Age, years	6.10 ± 3.24	5.83 ± 2.78	6.40 ± 1.96	0.11604	0.891	One-way ANOVA
Cumulative steroid duration, years	1.33 ± 0.89	0.77 ± 0.40	2.10 ± 1.20	7.29757	0.002	One-way ANOVA
Cumulative steroid dose, mg	5381.20 ± 2549.28	3400.67 ± 1229.12	6060.00 ± 2308.05	5.52756	0.007	One-way ANOVA
Steroid dose, mg/kg	259.57 ± 96.69	274.42 ± 293.92	329.00 ± 179.78	0.47270	0.626	One-way ANOVA
Calcium, mg/dL	9.52 ± 0.92	9.23 ± 0.90	9.50 ± 1.31	0.41431	0.663	One-way ANOVA
Phosphorus, mg/dL	5.71 ± 0.90	5.70 ± 0.81	5.72 ± 0.86	0.00160	0.998	One-way ANOVA
PTH, pg/mL	42.56 ± 19.30	42.93 ± 9.16	43.10 ± 14.31	0.00509	0.995	One-way ANOVA
ALP, U/L	120.40 ± 85.56	94.93 ± 18.70	122.60 ± 97.72	0.63347	0.535	One-way ANOVA
25-OH vitamin D, ng/mL	20.72 ± 6.64	22.73 ± 4.42	16.90 ± 7.16	2.69589	0.078	One-way ANOVA

Pearson correlation analysis showed a positive correlation between cumulative steroid duration and total dose (r = 0.564, p < 0.001), and between age and cumulative steroid dose (r = 0.660, p < 0.001; Table 4). There was a significant negative correlation between cumulative steroid duration and BMD Z-score (r = -0.339, p = 0.016), suggesting a duration-dependent effect of steroids on bone density. However, cumulative steroid dose did not correlate significantly with BMD Z-score (r = -0.130, p = 0.369).

**Table 4:** Correlation of age and steroid exposure with BMD BMD: bone mineral density

Variable 1	Variable 2	Pearson's r	t-value	p-value
Cumulative steroid duration, years	Cumulative steroid dose, mg	0.564	4.73	<0.001
Cumulative steroid duration, years	BMD Z-score	-0.339	-2.5	0.016
Cumulative steroid dose, mg	BMD Z-score	-0.130	-0.91	0.369
Age, years	Cumulative steroid duration, years	0.222	1.58	0.121
Age, years	Cumulative steroid dose, mg	0.660	6.09	<0.001

Ocular complications were observed in nine (18%) children: refractive errors in eight (16%), posterior subcapsular cataract in one (2%), and raised intraocular pressure in one (2%). Children with ocular complications were significantly older (9.13 vs 5.49 years, p = 0.004), had longer cumulative steroid duration (2.01 vs 1.14 years, p = 0.013), and higher cumulative steroid dose (6324 vs 4563 mg, p = 0.012), compared to those without ocular complications. Steroid dose per kg did not differ significantly between the two groups. No significant association was found between ocular complications and NS subtype or current disease activity.

There were no statistically significant differences in clinical and biochemical parameters between children in remission and those with active relapse (Table 5).

**Table 5:** Clinical and biochemical parameters by activity group Values are expressed as mean ± SD. Group 1: remission; Group 2: active relapse. p < 0.05 is considered statistically significant. ALP: alkaline phosphatase; PTH: parathormone

Variable	Group 1 (mean ± SD)	Group 2 (mean ± SD)	p-value	Statistical test
Cumulative years of steroid	1.27 ± 0.99	1.54 ± 0.79	0.08	Independent t-test
Cumulative steroid dose (mg)	4867.81 ± 2390.99	5173.33 ± 2465.85	0.72	Independent t-test
Total steroid dose (mg/kg)	288.36 ± 203.79	230.32 ± 95.70	0.13	Independent t-test
Age (years)	5.89 ± 2.64	6.94 ± 3.71	0.52	Independent t-test
Calcium (mg/dL)	9.50 ± 1.03	9.11 ± 0.78	0.30	Independent t-test
Phosphorus (mg/dL)	5.70 ± 0.87	5.77 ± 0.79	0.88	Independent t-test
PTH (pg/mL)	42.63 ± 14.72	43.44 ± 20.28	0.86	Independent t-test
ALP (U/L)	110.49 ± 69.53	125.56 ± 99.17	0.99	Independent t-test
25-OH vitamin D (ng/mL)	20.93 ± 6.22	18.89 ± 7.22	0.28	Independent t-test

## Discussion

The demographic profile, with male predominance (66%) and mean age around six years, is consistent with published data on childhood NS [10,11]. FRNS formed the largest subgroup (50%), followed by IFRNS (30%) and SDNS (20%), reflecting typical patterns in steroid-responsive disease. In this cohort of children with SSNS on prolonged corticosteroid therapy, we observed a high prevalence of subclinical bone loss and a notable burden of ocular complications.

Half of the children had osteopenia and 6% had osteoporosis on DXA, indicating that clinically silent skeletal compromise is common in this population. The significant association between steroid duration and lower BMD and the correlation between cumulative exposure and BMD Z-score support a duration- and dose-dependent glucocorticoid effect on bone [12,13]. Elevated PTH and ALP, along with low vitamin D in children with osteoporosis, suggest secondary hyperparathyroidism and increased bone turnover, while normal serum calcium likely reflects homeostatic compensation [14,15]. These findings align with reports of glucocorticoid-induced impairment of osteoblast function and reduced calcium absorption in children receiving long-term steroids [16,17].

The mechanisms underlying glucocorticoid-induced bone loss are multifactorial. Corticosteroids directly inhibit osteoblast differentiation and function, promote osteoblast and osteocyte apoptosis, and prolong osteoclast survival [18]. They also reduce intestinal calcium absorption and increase renal calcium excretion, leading to secondary hyperparathyroidism [19]. In children, the effects are compounded by interference with growth plate function and pubertal bone accrual [20]. Our finding of higher PTH and ALP in children with osteoporosis supports this pathophysiological cascade.

Several studies have reported a similar prevalence of reduced BMD in children with NS. A multicenter study by Soliman et al. found osteopenia in 48% and osteoporosis in 12% of children on prolonged steroids [6], comparable to our findings. Jesmin et al. reported reduced BMD in 52% of children with relapsing NS [15], while El-Mashad et al. found abnormal BMD in 56% [17]. The consistency across studies underscores the magnitude of this problem. Our observation that steroid duration correlated more strongly with BMD than cumulative dose aligns with findings by Yadav et al. [18], suggesting that sustained exposure may be more detrimental than intermittent high-dose therapy.

Children with SDNS had the greatest steroid burden and tended to have lower vitamin D levels, placing them at higher risk for skeletal complications. Vitamin D deficiency is prevalent in children with NS, attributed to urinary losses of vitamin D-binding protein during relapse and inadequate supplementation [21,22]. The lower vitamin D in SDNS children, though not statistically significant, may reflect cumulative urinary losses and warrants targeted supplementation strategies. These observations strengthen the case for integrating routine BMD assessment and vitamin D evaluation into the long-term follow-up of SDNS and other high-exposure phenotypes.

Ocular morbidity was detected in 18% of children, predominantly as refractive errors (16%), which were significantly associated with both duration and cumulative dose of steroids. Although posterior subcapsular cataract and raised intraocular pressure were infrequent, their occurrence in heavily treated children underscores the need for systematic screening. Steroid-induced changes in lens metabolism and aqueous humor dynamics are recognized mechanisms for cataract and glaucoma [23]. Corticosteroids alter lens fiber protein synthesis and increase osmotic stress, leading to posterior subcapsular opacities [24]. They also reduce aqueous outflow facility by increasing extracellular matrix deposition in the trabecular meshwork, elevating intraocular pressure [7].

The prevalence of refractive errors in our study is consistent with reports by Kyrieleis et al., who found refractive changes in 15%-20% of children on chronic steroids [23]. The low prevalence of cataract (2%) may reflect early detection and intervention, shorter follow-up duration, or protective factors such as younger age at initiation. Posterior subcapsular cataract has been reported in 10%-30% of patients on long-term steroids, with risk increasing with cumulative dose beyond 10 g prednisolone equivalent [24]. Our cohort's mean cumulative dose of approximately 5 g may explain the lower cataract prevalence, though longer follow-up might reveal increasing rates.

Regular ophthalmologic surveillance, early detection of refractive errors, and timely management of cataract or glaucoma are essential components of care. The American Academy of Pediatrics recommends baseline and periodic ophthalmologic examination for children on chronic corticosteroids, though specific intervals remain unstandardized [7]. Our findings support annual screening in children with cumulative exposure exceeding one year or total dose exceeding 5 g prednisolone equivalent.

The strengths of this study include standardized DXA-based BMD assessment, comprehensive biochemical profiling, and systematic ophthalmologic evaluation in a well-defined SSNS cohort. The cross-sectional design allowed efficient characterization of complications across disease subtypes. This study has several limitations. Its single-center design and modest sample size may have limited the statistical power to detect weaker associations, particularly for less common outcomes such as osteoporosis and ocular complications. Dietary intake, including calcium and vitamin D consumption, was not quantitatively assessed and may have acted as an unmeasured confounder. The cross-sectional design precludes causal inference and does not allow assessment of the temporal evolution of these complications or their response to interventions. Nevertheless, the high prevalence of osteopenia and clinically relevant ocular abnormalities observed in our cohort underscores the importance of proactive bone and ophthalmologic monitoring in children with SSNS receiving prolonged corticosteroid therapy.

Future research should focus on prospective longitudinal studies examining the natural history of bone and ocular complications, optimal screening intervals, and efficacy of preventive interventions. Randomized trials evaluating vitamin D and calcium supplementation protocols, as well as steroid-sparing agents in high-risk phenotypes, are needed. Cost-effectiveness analyses of routine DXA screening in resource-limited settings would inform evidence-based guidelines. Collaboration across centers would enable larger cohorts to define risk stratification.

## Conclusions

Children with SSNS receiving prolonged corticosteroid therapy have a high prevalence of reduced BMD and ocular complications. Longer duration of steroid therapy was associated with lower BMD, while refractive errors were the most common ocular abnormality. These findings support the need for regular bone health and ophthalmologic monitoring in children receiving long-term corticosteroid therapy.
